# Artificial intelligence and infodemic management: a governance framework for digital public health decision-making

**DOI:** 10.3389/fdgth.2026.1830005

**Published:** 2026-09-09

**Authors:** Angelo Cianciulli, Emanuela Santoro, Antonietta Pacifico, Roberta Manente, Marika Finizio, Giuseppina Speziga, Mario Capunzo, Giovanni Boccia

**Affiliations:** 1 Department of Medicine, Surgery and Dentistry “Scuola Medica Salernitana”, University of Salerno, Salerno, Italy; 2 San Giovanni di Dio e Ruggi D’Aragona University Hospital, Salerno, Italy; 3 Integrated Care Department of Health Hygiene and Evaluative Medicine, San Giovanni di Dio e Ruggi D’Aragona University Hospital, Salerno, Italy; 4 Hospital and Epidemiological Hygiene Unit, San Giovanni di Dio and Ruggi D’Aragona University Hospital, Salerno, Italy

**Keywords:** artificial intelligence, digital surveillance, health misinformation, infodemic, policy translation, public health governance

## Abstract

**Background:**

Digital communication ecosystems have profoundly transformed the circulation of health information. During public health crises, large volumes of information—including misinformation and disinformation—can spread rapidly across online platforms, generating complex information environments commonly described as infodemics. These dynamics can influence public risk perception, undermine trust in health institutions, and affect adherence to preventive measures. At the same time, advances in artificial intelligence (AI) and digital technologies have created new opportunities for monitoring information flows and identifying emerging misinformation patterns in real time. However, the translation of AI-generated insights into coordinated public health decision-making processes remains limited.

**Methods:**

This study developed a conceptual governance framework aimed at explaining how AI-generated insights derived from digital information ecosystems can be translated into evidence-informed public health responses. The framework was developed through a secondary conceptual analysis of the complete evidence extraction database generated from a previously published scoping review including 63 studies. The methodological approach combined thematic synthesis of empirical evidence with conceptual modeling techniques commonly used in public health systems research.

**Results:**

The synthesis of the literature identified several thematic domains describing how digital technologies and artificial intelligence are used to monitor and respond to health misinformation within digital communication ecosystems. Building on these domains, this study proposes a governance-oriented conceptual framework integrating digital data ecosystems, artificial intelligence analytics, interpretive public health expertise, and institutional governance mechanisms.

**Conclusions:**

Artificial intelligence technologies offer powerful tools for monitoring complex digital information environments, yet their integration into public health decision-making requires governance models capable of linking analytical insights with institutional responses. The proposed framework contributes to the emerging field of digital public health governance by providing a structured conceptual model for translating AI-generated insights into coordinated public health actions during infodemic events. Although the framework remains preliminary and requires formal validation, it provides a structured conceptual basis for future empirical implementation and governance research.

## Introduction

The rapid expansion of digital communication technologies has profoundly transformed the ways in which health information is produced, disseminated, and consumed. Online platforms, social media networks, search engines, and digital communication systems have become central channels through which individuals access information related to health risks, disease prevention, and medical interventions. While these technologies have significantly improved the speed and reach of health communication, they have also created complex information ecosystems in which accurate information coexists with misinformation and disinformation.

During major public health crises, the proliferation of conflicting and misleading information can generate what the World Health Organization has described as an infodemic, defined as an overabundance of information, both accurate and false, that spreads rapidly during disease outbreaks and makes it difficult for individuals to identify trustworthy sources of guidance (1). Infodemics represent a significant challenge for public health systems because they can influence risk perception, undermine trust in health institutions, and affect adherence to recommended health behaviors (2).

The COVID-19 pandemic provided a clear illustration of the scale and impact of contemporary infodemics. The global spread of misinformation related to disease transmission, preventive measures, and vaccine safety created complex information environments in which scientific evidence often competed with misleading narratives circulating across digital platforms. Studies examining online communication dynamics during the pandemic have demonstrated that social media networks can amplify misinformation at unprecedented speed and scale, facilitating the rapid diffusion of misleading health narratives across digital communities (3, 4). These dynamics have important implications for public health responses, as misinformation can influence population behavior, reduce compliance with preventive measures, and increase vaccine hesitancy.

The increasing availability of large-scale digital data has stimulated the emergence of digital epidemiology, a field that integrates digital data streams with traditional epidemiological approaches in order to monitor population health dynamics and behavioral trends (5). Digital epidemiology leverages information generated through online platforms, search queries, and social media interactions to detect signals related to disease outbreaks, risk perception, and population behavior. Systematic reviews have shown that internet-based data sources can complement traditional surveillance systems by providing real-time insights into health-related information flows and emerging public health concerns (6).

At the same time, advances in artificial intelligence (AI) and machine learning technologies have expanded the analytical capabilities available for analyzing complex digital information environments. Artificial intelligence methods—including natural language processing, sentiment analysis, and automated misinformation detection—enable large-scale analysis of unstructured digital data, allowing researchers and public health authorities to identify emerging narratives, detect misinformation patterns, and monitor public sentiment during health crises (7). These technologies are increasingly viewed as key tools for strengthening public health intelligence systems and improving situational awareness during rapidly evolving public health emergencies.

The convergence of digital data and artificial intelligence has also been conceptualized within broader discussions regarding the transformation of healthcare systems through advanced computational technologies. The integration of AI with medical expertise has been described as a critical component of future healthcare systems capable of combining large-scale data analysis with clinical and public health knowledge (8). Within public health contexts, such technological developments have opened new opportunities for monitoring information dynamics and identifying emerging threats within digital communication ecosystems.

Despite the growing availability of digital surveillance tools and AI-driven analytical methods, the translation of technological insights into coordinated public health responses remains limited. Many digital monitoring initiatives operate primarily within research environments or pilot projects, while institutional mechanisms capable of integrating digital analytics into public health decision-making processes remain underdeveloped. This gap reflects broader governance challenges related to the integration of digital technologies within public health systems.

The need for effective governance models has been highlighted in discussions on governance for health, which emphasize the importance of coordinated institutional frameworks capable of integrating evidence, policy development, and public health action across complex systems (9). Implementation research further underscores the importance of institutional mechanisms that facilitate the translation of knowledge and evidence into effective policy interventions (10). Within the context of digital public health, these governance challenges become even more complex due to the rapid evolution of technological systems and the transnational nature of digital communication environments.

Recent research has begun to explore the role of artificial intelligence and digital technologies in addressing health misinformation and infodemics. A growing body of literature examines the use of AI-driven tools for detecting misinformation, monitoring online discourse, and supporting public health communication strategies. In particular, our previously published scoping review examining 63 studies on artificial intelligence and digital technologies used to address health misinformation and infodemics synthesized the current evidence base and identified key operational domains supporting digital public health responses (11). These studies highlight the increasing importance of digital tools in the monitoring and management of complex information ecosystems.

However, while existing research has focused primarily on technological solutions for detecting misinformation and monitoring information flows, comparatively less attention has been devoted to the governance structures required to translate these analytical insights into coordinated public health responses. The management of infodemics requires not only technological capabilities but also institutional mechanisms capable of integrating digital analytics with decision-making processes, communication strategies, and regulatory interventions.

Addressing this gap requires conceptual frameworks capable of linking digital information ecosystems, artificial intelligence analytics, human expertise, and institutional governance mechanisms within coherent public health systems. Understanding how insights derived from digital surveillance systems can inform public health decision-making represents a critical challenge for contemporary public health governance.

In particular, the present study does not constitute an update of the previous scoping review but a secondary conceptual analysis of its complete evidence base. Whereas the scoping review was designed to identify, categorize, and synthesize the available evidence on AI and digital technologies for infodemic management, the present work develops a novel governance-oriented conceptual framework by integrating those empirical findings with governance theory and public health systems principles. Accordingly, the originality of this manuscript lies in the generation of new conceptual relationships and governance propositions that were not reported in the preceding review (11).

Despite the growing body of research examining the role of artificial intelligence in detecting and analyzing online misinformation, less attention has been devoted to understanding how insights generated from digital information ecosystems can be effectively integrated into public health governance structures. In particular, there remains a need for analytical models capable of explaining how digital signals identified through AI-driven monitoring systems may be translated into coordinated institutional responses within public health systems. To address this gap, the present study develops a governance-oriented conceptual framework designed to link digital data ecosystems, artificial intelligence analytics, interpretive expertise, and institutional decision-making processes in the management of infodemics.

The aim of this study was to develop a conceptual governance framework explaining how artificial intelligence–driven insights derived from digital information ecosystems can be translated into coordinated public health responses to infodemics.

## Methods

### Study design and conceptual approach

This study adopted a conceptual framework development approach grounded in evidence synthesis and theoretical integration. Conceptual frameworks are widely used in public health and health systems research to synthesize empirical evidence and theoretical constructs into structured analytical models capable of guiding policy development, research agendas, and implementation strategies (12, 13). Within complex health systems, such frameworks are particularly useful for integrating heterogeneous forms of evidence and for clarifying the relationships between technological innovations, institutional processes, and population health outcomes.

The objective of the present study was not to test causal hypotheses but to develop a governance-oriented analytical framework capable of explaining how artificial intelligence–driven insights derived from digital information ecosystems can be translated into coordinated public health responses during infodemic events. The methodological strategy followed an evidence-informed conceptual synthesis process integrating findings from the literature with theoretical considerations derived from digital public health and health governance research.

### Evidence base

The empirical foundation of the conceptual framework derives from evidence synthesized in a previously conducted scoping review examining the role of artificial intelligence and digital technologies in addressing health misinformation and infodemics (11). The scoping review followed methodological guidance provided by the PRISMA extension for scoping reviews (PRISMA-ScR) and adhered to established standards for evidence synthesis in health research (14).

The review examined studies investigating digital surveillance systems, social media analytics, machine learning models, and natural language processing techniques used to monitor and analyze health-related information circulating across digital communication platforms. The literature search included studies focusing on the use of artificial intelligence for detecting misinformation, monitoring public sentiment, and supporting public health communication strategies in digital environments.

Previous research has shown that digital information ecosystems provide valuable sources of data for understanding the dynamics of health communication and information diffusion during public health crises (6, 15). These digital data streams include social media interactions, online search behavior, digital news dissemination, and communication patterns within online communities. Within this context, artificial intelligence technologies have increasingly been applied to analyze large volumes of unstructured data generated across digital communication networks (7).

The evidence extracted from the scoping review was analyzed to identify recurring thematic domains relevant to the development of the governance framework. These domains were subsequently synthesized through an interpretive analytical process in order to construct the conceptual architecture of the proposed model, linking empirical evidence on digital infodemic responses with a governance-oriented analytical structure for public health decision-making.

The analytical dataset used for framework development consisted of the complete evidence extraction database generated during the previously published scoping review, including all 63 studies that met the eligibility criteria. Rather than relying exclusively on the published narrative synthesis, the present conceptual analysis re-examined the study-level extraction records to identify recurrent operational functions, governance mechanisms, implementation characteristics, and public health applications. The unit of analysis was therefore represented by the conceptual and operational elements reported across the included studies that were relevant to the governance of AI-enabled infodemic management.

### Framework development process

The conceptual framework was developed through an iterative, evidence-informed synthesis process designed to translate the findings of the scoping review into a structured governance model for AI-enabled public health responses to infodemics. The purpose of this process was not to generate a new empirical classification of studies, but to identify the main functional domains emerging from the evidence base and to organize them into an analytically coherent framework capable of explaining how digital information signals may be transformed into institutional public health action.

The framework development process followed three sequential analytical stages. To enhance transparency and reproducibility, the framework development process was informed by established approaches to evidence-informed conceptual model construction described by Jabareen and subsequent health systems framework development literature. The analytical progression from evidence synthesis to conceptual model development followed a structured sequence involving (1) identification of recurring functional domains, (2) thematic aggregation into higher-order conceptual categories, (3) examination of logical relationships between categories, and (4) integration into a governance-oriented systems architecture. The framework therefore represents an evidence-informed analytical abstraction derived from the reviewed literature rather than a normative or empirically validated operational model. In the first stage, the evidence extracted from the scoping review was re-examined through a thematic interpretive lens in order to identify recurring operational functions associated with digital responses to health misinformation. Particular attention was given to the role played by digital platforms as sources of information signals, the use of artificial intelligence techniques for information analysis, the contribution of expert interpretation in contextualizing computational outputs, and the institutional mechanisms through which such insights may support public health responses. This stage was primarily inductive, as the functional domains were allowed to emerge from the reviewed evidence rather than being imposed *a priori*. Nevertheless, during the subsequent stages of conceptual integration, governance and health systems concepts derived from the broader literature were incorporated deductively to organize the emerging domains into a coherent governance-oriented architecture.

Framework development was undertaken collaboratively by the research team, whose expertise encompassed public health, epidemiology, digital health, evidence synthesis, and health governance. The analytical process was discussion-based and consensus-oriented, with iterative meetings conducted to refine conceptual domains and their relationships. Because the objective was conceptual framework development rather than formal qualitative research, no independent duplicate coding procedure or qualitative data analysis software was used. The emphasis was placed on conceptual coherence, recurrence across the evidence base, and governance relevance rather than quantitative coding frequency.

In the second stage, the recurring functions identified across the included studies were grouped into broader conceptual domains reflecting sequential processes within digital public health decision-making. This grouping process was informed by analytical coherence and by the need to distinguish between technological, interpretive, and institutional components of infodemic management. The aim of this phase was to move from heterogeneous empirical findings to a higher-order conceptual structure capable of representing the transition from digital information environments to public health action.

In the third stage, the conceptual domains derived from the evidence synthesis were integrated into a multi-layer governance architecture. During this stage, the emphasis was placed on identifying the logical relationships between the different domains and on clarifying the interfaces between data generation, computational analysis, expert interpretation, and institutional governance. This process led to the development of a four-component framework composed of digital data ecosystems, artificial intelligence analytics, interpretive public health expertise, and institutional governance mechanisms.

The selection of these four components was based on their recurrence across the evidence base and on their conceptual relevance to the governance of infodemics in digital environments. Rather than representing isolated categories, these components were conceptualized as interacting dimensions of a socio-technical public health system. The resulting framework therefore reflects both the empirical patterns identified in the scoping review and the governance-oriented analytical perspective adopted in the present study.

This analytical approach is consistent with conceptual framework development methods used in health systems and implementation research, where empirical evidence is synthesized and abstracted into structured models capable of informing research, policy, and practice (10, 12, 13). The thematic domains identified through this interpretive synthesis are presented in the Results section and provide the evidence-informed basis for the conceptual governance framework proposed in this study. Importantly, while the scoping review provided the empirical evidence base for identifying relevant themes and operational functions, the present study extends beyond descriptive synthesis by integrating these findings into a structured governance-oriented conceptual model. The resulting architecture comprises four sequential operational components (Data Ecosystem, AI/ML Analytics, Interpretation and Translation, and Decision and Policy), supported by a transversal governance layer encompassing equity, ethics, transparency, accountability, and legal-regulatory oversight. Rather than representing a fifth operational component, this governance layer functions as a cross-cutting enabling dimension that informs and supports all stages of the framework. The aim of this analytical step is to translate heterogeneous evidence on digital infodemic responses into a coherent framework capable of explaining how AI-generated insights may inform institutional public health decision-making processes.

## Results

### Evidence synthesis

The synthesis of the literature identified several thematic domains describing how digital technologies and artificial intelligence are used to monitor and respond to health misinformation within digital communication ecosystems. These domains reflect the main functional areas emerging from the evidence base and provide the empirical foundation for the conceptual framework developed in this study. The thematic domains identified through the evidence synthesis are summarized in Table 1 together with their corresponding evidence base. Their subsequent translation into the four governance components of the proposed conceptual framework is presented in Table 2.

**Table 1:** Evidence domains informing the development of the conceptual governance framework.

Evidence domain	Type of evidence	Description	Representative studies
Digital monitoring and infoveillance	Empirical studies	Studies examining the use of digital data sources such as social media platforms, search engines, and online communication networks to monitor the circulation of health information and detect early signals of misinformation within digital environments.	(5, 6, 15)
Artificial intelligence for misinformation detection	Empirical studies	Studies applying machine learning algorithms, natural language processing, and computational analytics to identify misinformation patterns, analyze information diffusion, and monitor public sentiment within digital communication ecosystems.	(7, 11)
Infodemic dynamics in digital ecosystems	Empirical studies	Studies analyzing the mechanisms through which misinformation spreads across social media platforms and digital communication networks during public health crises, including the amplification of misleading narratives and the formation of polarized information environments.	(3, 4, 16)
Public health communication and response strategies	Empirical studies	Studies examining digital communication interventions, risk communication strategies, and institutional responses designed to mitigate the impact of misinformation and support evidence-based public health messaging.	(2, 17)
Governance, ethics, and regulatory frameworks	Policy and theoretical literature	Studies addressing governance models, ethical considerations, and regulatory frameworks guiding the use of artificial intelligence and digital technologies in healthcare and public health systems.	(17–21)

The table summarizes the principal thematic domains identified during the conceptual synthesis rather than reporting quantitative frequencies, since individual studies frequently contributed to multiple domains simultaneously.

**Table 2:** Translation of evidence domains into governance framework components.

Evidence domain	Main analytical contribution	Governance concept integrated	Final framework component
Digital monitoring and infoveillance	Digital data sources generate real-time signals	Public health intelligence	Data Ecosystem
Artificial intelligence for misinformation detection	Computational analysis	Human oversight	AI/ML Analytics
Infodemic dynamics in digital ecosystems	Information propagation	Contextual interpretation	Interpretation & Translation
Public health communication and response strategies	Institutional communication	Decision support	Decision & Policy
Governance, ethics, and regulatory frameworks	Accountability and oversight	Cross-cutting governance principles	Governance Panel

A central finding of the literature concerns the increasing importance of digital communication platforms as environments in which health information and misinformation circulate. Social media platforms, search engines, messaging applications, and digital news systems have become primary channels through which individuals access and share health-related information. During public health emergencies, these platforms generate large volumes of user-generated content reflecting public perceptions, attitudes, and behaviors related to health risks. Studies examining communication dynamics during the COVID-19 pandemic have demonstrated that digital networks can rapidly amplify both accurate information and misleading narratives, contributing to complex information environments characterized by high levels of uncertainty and competing interpretations of scientific evidence (3, 4).

The growing availability of digital communication data has stimulated the development of digital epidemiology, a field that integrates digital data streams with epidemiological methods in order to monitor population behavior and emerging public health threats. Digital epidemiology approaches use data generated through online platforms, search queries, and social media interactions to detect patterns in health-related communication and identify signals related to disease outbreaks or changes in public risk perception (5). Systematic reviews examining digital surveillance systems have shown that internet-based data sources can complement traditional public health monitoring systems by providing real-time insights into communication dynamics and population behavior (6).

Advances in artificial intelligence and machine learning have further expanded the analytical capabilities available for examining digital information environments. AI-driven approaches enable large-scale analysis of heterogeneous digital datasets through techniques such as natural language processing, topic modeling, and sentiment analysis. These methods have been applied to detect misinformation patterns, track the diffusion of health narratives across digital networks, and analyze public attitudes toward preventive health measures and vaccination campaigns (7, 8). Within this context, artificial intelligence has increasingly been proposed as a tool capable of supporting public health surveillance and communication strategies by identifying emerging information risks within digital ecosystems.

Recent scoping reviews examining the use of artificial intelligence and digital technologies in public health have highlighted the growing role of computational tools in responding to health misinformation. Evidence suggests that AI-driven systems can support the detection of misleading narratives, facilitate monitoring of online discourse, and inform communication strategies designed to counter misinformation (11). However, the literature also emphasizes that technological innovation alone cannot ensure effective responses to infodemics.

A recurring theme across the evidence synthesis concerns the gap between technological capabilities and institutional decision-making processes. While digital surveillance systems and artificial intelligence tools can generate large volumes of analytical insights regarding information dynamics, public health institutions often lack governance mechanisms capable of translating these insights into coordinated policy responses. Governance frameworks within public health systems were originally designed to operate within traditional information environments and may not be fully adapted to the complexities of digital communication ecosystems (9).

Implementation research has shown that effective translation of knowledge into policy requires institutional mechanisms capable of integrating scientific evidence with decision-making processes and operational interventions (10). Within the context of digital public health, this challenge becomes even more complex due to the speed and scale of information diffusion across online platforms.

These thematic domains provided the analytical basis for the development of the governance framework presented in the following section. The analysis of the literature therefore suggests that effective responses to infodemics require not only technological tools capable of monitoring digital information ecosystems but also governance structures capable of integrating digital analytics, expert interpretation, and institutional decision-making processes.

Unlike the preceding scoping review, which identified and categorized the evidence, the present analytical process synthesized these domains into an integrated governance architecture describing their functional relationships within public health decision-making. Table 2 illustrates the analytical progression from the evidence domains identified in the scoping review to the conceptual components of the proposed governance framework. While the empirical evidence informed the identification of the principal functional domains, governance theory and public health systems concepts were subsequently integrated to organize these domains into a coherent governance-oriented architecture.

### Governance framework architecture

Building on the evidence synthesis, the present study developed a conceptual governance framework designed to explain how insights generated through artificial intelligence–driven analysis of digital information ecosystems can be translated into coordinated public health responses. The proposed framework conceptualizes infodemic management as a socio-technical governance system in which technological infrastructures, analytical tools, human expertise, and institutional decision-making mechanisms interact within an integrated public health response structure.

**Figure 1:**
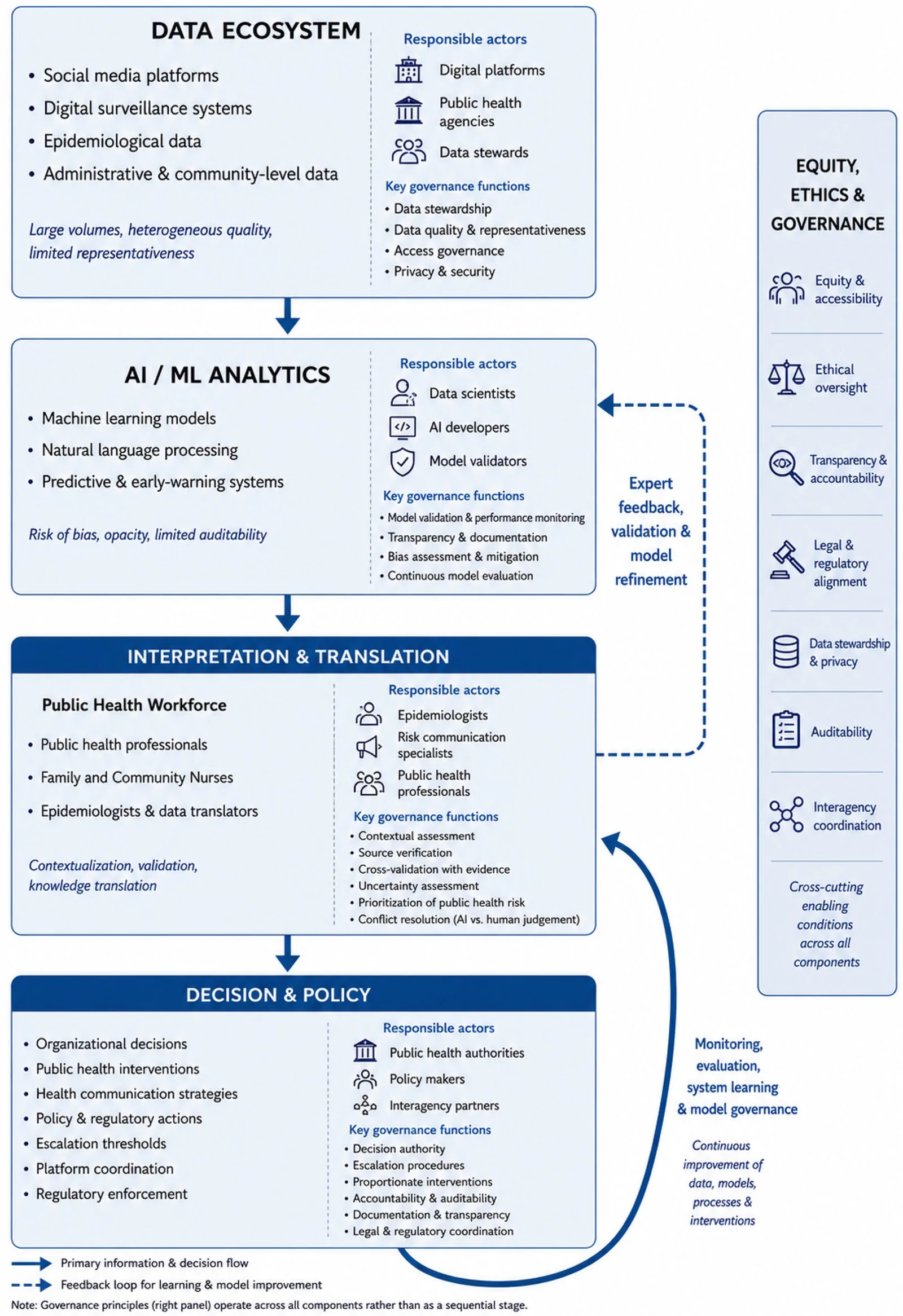
Governance architecture for AI-driven public health responses to infodemics.

Although the empirical evidence synthesized in the underlying scoping review primarily focused on digital communication ecosystems, the framework intentionally incorporates epidemiological and administrative data because public health decision-making routinely relies on the integration of digital intelligence with conventional surveillance systems, health service indicators, and administrative information. Their inclusion reflects the broader governance perspective adopted in this study rather than an expansion of the original evidence base. Accordingly, the governance panel illustrated in Figure 1 should not be interpreted as a fifth operational component. Rather, it represents a transversal enabling layer that provides ethical, legal, and governance principles supporting all four sequential components of the framework throughout the decision-making process.

The first structural component of the framework corresponds to the digital data ecosystem, which represents the information environment in which contemporary infodemics emerge and evolve. Digital communication platforms produce vast volumes of real-time information reflecting population perceptions, beliefs, and behaviors related to health risks. These digital traces constitute valuable sources of data for monitoring communication dynamics and identifying emerging misinformation patterns during public health crises.

The second component of the framework involves artificial intelligence analytics, which enable the large-scale analysis of digital information environments. Machine learning algorithms and natural language processing techniques allow researchers and public health authorities to process large volumes of unstructured data and identify patterns within complex communication networks. These analytical tools support the detection of misinformation narratives, monitoring of information diffusion dynamics, and analysis of public sentiment related to health interventions.

The third component of the framework corresponds to interpretive expertise, which represents the role of public health professionals in contextualizing algorithmic outputs and translating analytical insights into meaningful knowledge for decision-making processes. Human expertise remains essential for evaluating the reliability of digital signals, interpreting analytical findings, and integrating technological insights with epidemiological knowledge and public health practice. Within the proposed framework, public health professionals operate through a structured human-in-the-loop validation process aimed at assessing the contextual validity of AI-generated outputs, identifying potential false-positive and false-negative classifications, verifying source credibility, and evaluating the public health relevance of detected narratives before their translation into institutional actions.

The final component of the framework consists of institutional governance mechanisms within public health systems. These mechanisms include communication strategies, regulatory interventions, and coordinated policy responses aimed at mitigating the impact of misinformation and strengthening the resilience of public health systems in digital communication environments. An additional governance function of the interpretive layer consists of providing systematic feedback to the AI analytics component. Cases identified by public health professionals as algorithmic misclassifications, ambiguous narratives, contextual misunderstandings, or partially accurate information (“half-truths”) may be incorporated into expert-annotated datasets used for model calibration, retraining, and performance improvement. This feedback mechanism supports continuous learning and adaptive refinement of AI-driven surveillance systems, strengthening their ability to detect complex forms of health misinformation over time.

Across these four components, the framework emphasizes the importance of governance conditions capable of ensuring responsible and effective use of artificial intelligence technologies. Ethical considerations, regulatory frameworks, and institutional accountability mechanisms play a critical role in ensuring that AI-driven surveillance systems operate within transparent and socially responsible governance structures (18, 19). Concerns related to algorithmic bias and social inequality further highlight the need for governance models capable of ensuring fairness and equity in the deployment of digital health technologies (20, 21).

Within the proposed framework, the transition between layers reflects a sequential process through which digital information signals are progressively transformed into public health knowledge and institutional action. Digital information ecosystems generate large volumes of user-generated content and communication signals that may contain both accurate information and misinformation. Artificial intelligence analytics are applied to these data streams to detect patterns, identify misinformation narratives, and monitor the dynamics of information diffusion across digital platforms. The outputs generated by these analytical systems require expert interpretation by public health professionals, who contextualize computational findings within epidemiological, behavioral, and communication frameworks. Finally, these interpreted insights inform institutional governance processes, enabling public health authorities to design communication strategies, coordinate responses with digital platforms, and implement evidence-informed interventions aimed at mitigating the spread and impact of health misinformation.

The conceptual architecture of the governance framework developed from this study is illustrated in Figure 1, which depicts the interaction between digital data ecosystems, artificial intelligence analytics, interpretive expertise, and institutional governance mechanisms within public health systems.

The solid arrows represent the primary progression from digital information generation to institutional public health action. The dashed arrows indicate continuous feedback mechanisms through which expert validation informs AI model refinement and governance learning. The governance panel represents cross-cutting enabling conditions—including ethics, transparency, accountability, and equity—that operate across all framework components rather than as a sequential stage.

### Governance functions across framework components

Beyond representing a sequential analytical process, each component of the proposed framework performs distinct governance functions. Within the Data Ecosystem layer, governance responsibilities include data stewardship, assessment of data quality and representativeness, management of data access, and protection of privacy. The AI/ML Analytics layer is responsible for algorithm validation, transparency, performance monitoring, documentation of analytical outputs, and continuous model evaluation. The Interpretation and Translation layer provides contextual verification, evidence appraisal, uncertainty assessment, prioritization of detected signals, and resolution of discrepancies between computational outputs and expert judgement. Finally, the Decision and Policy layer encompasses institutional authority, escalation procedures, implementation of proportional public health interventions, communication strategies, interagency coordination, and accountability for policy decisions. Across all layers, equity, ethics, transparency, and legal oversight operate as enabling governance conditions supporting responsible use of AI within public health systems.

## Discussion

The findings of this study highlight the growing importance of digital information ecosystems as determinants of public health outcomes in contemporary societies. Building on the evidence synthesized in the previous scoping review, the present study extends the existing literature by translating empirical findings into a governance-oriented conceptual framework. While prior research has primarily focused on the development of technological tools for detecting and monitoring health misinformation, the framework proposed here emphasizes the institutional and governance dimensions required to transform digital intelligence into coordinated public health action. By integrating digital data ecosystems, artificial intelligence analytics, interpretive public health expertise, and institutional governance mechanisms into a unified analytical structure, this study contributes to clarifying how AI-generated insights may inform decision-making processes within public health systems.

The rapid expansion of digital communication technologies has created complex information environments in which health information circulates at unprecedented speed and scale. Within these environments, misinformation and disinformation can spread rapidly across digital networks, influencing population behavior and shaping public perceptions of health risks. The emergence of infodemics has therefore introduced new challenges for public health systems that were traditionally designed to operate within more controlled communication environments. From a public health governance perspective, the proposed framework also highlights the importance of integrating digital intelligence capabilities within institutional decision-making structures. The capacity to monitor digital information ecosystems through AI-driven analytics is only effective when supported by governance mechanisms capable of interpreting signals, coordinating responses, and translating digital insights into risk communication strategies and policy actions. In this sense, infodemic management should be understood not only as a technological challenge but also as a governance and institutional coordination issue within modern public health systems.

The framework proposed in this study also contributes to the broader literature on infodemic management and digital public health governance. Unlike existing infodemic management frameworks, which primarily focus on monitoring activities, communication responses, or operational guidance, the present model explicitly conceptualizes the governance pathway through which AI-generated digital intelligence is translated into institutional decision-making. The framework therefore contributes a governance-oriented perspective that integrates technological, interpretive, and policy dimensions within a single socio-technical architecture. Existing approaches, particularly those developed by the World Health Organization, have emphasized the importance of monitoring information environments, strengthening risk communication, and coordinating multi-sector responses to misinformation. While these approaches provide important operational guidance, the model presented in this study complements them by explicitly integrating digital data ecosystems, artificial intelligence analytics, expert interpretation, and institutional governance within a single analytical structure. In this sense, the proposed framework does not replace existing infodemic management strategies but rather provides a conceptual lens for understanding how digital intelligence can be translated into coordinated public health decision-making processes.

The conceptual framework developed in this study contributes to the growing literature examining the intersection between digital technologies, artificial intelligence, and public health governance. By integrating insights from digital epidemiology, artificial intelligence research, and health systems governance literature, the proposed model provides an analytical structure capable of explaining how digital information flows can be transformed into coordinated public health responses.

A central insight emerging from the evidence synthesis concerns the increasing role of digital data ecosystems as sources of public health intelligence. Digital communication platforms generate large volumes of real-time information reflecting public perceptions, beliefs, and behaviors related to health risks. Previous research has demonstrated that such data streams can provide valuable insights into emerging health threats, public attitudes toward preventive measures, and patterns of misinformation diffusion within online communities (5, 6). The integration of these digital signals within public health surveillance systems represents a significant opportunity for improving situational awareness during health crises.

Artificial intelligence technologies have further expanded the analytical capabilities available for examining digital communication environments. Machine learning algorithms and natural language processing techniques enable large-scale analysis of unstructured data generated across digital platforms, allowing researchers and public health authorities to detect misinformation patterns, identify emerging narratives, and monitor public sentiment toward health interventions (7, 8). In this sense, artificial intelligence can function as a form of computational public health intelligence, capable of supporting early detection of information risks within digital ecosystems.

However, the findings of this study also highlight an important limitation in current approaches to digital infodemic management. While technological tools for monitoring digital communication environments have rapidly advanced, institutional governance mechanisms capable of translating analytical insights into coordinated public health responses remain underdeveloped. This gap reflects broader structural challenges within public health systems related to the integration of digital technologies into institutional decision-making processes.

The framework proposed in this study conceptualizes infodemic management not simply as a technological challenge but as a governance problem requiring coordination between technological infrastructures, analytical expertise, and institutional decision-making mechanisms. Governance for health approaches emphasize the importance of collaborative institutional arrangements capable of integrating scientific evidence, policy development, and public health action across complex systems (9). Within digital communication environments, such governance mechanisms must also incorporate the analytical capabilities provided by artificial intelligence and digital surveillance systems.

Implementation research has shown that the translation of knowledge into effective public health interventions requires institutional processes capable of linking evidence generation with decision-making and operational implementation (10). In the context of digital infodemics, this translation process involves transforming insights generated through digital surveillance and artificial intelligence analysis into targeted communication strategies, regulatory interventions, and policy responses aimed at mitigating misinformation and strengthening public trust in health institutions. Depending on the characteristics and severity of the detected signal, governance responses may include targeted risk communication, prebunking strategies, correction campaigns, collaboration with digital platforms, adaptation of health services, regulatory actions, or coordinated interagency responses. The selection of interventions should be proportionate to the assessed public health risk and supported by transparent governance procedures.

Another important dimension emerging from the analysis concerns the ethical and governance challenges associated with the use of artificial intelligence technologies in public health contexts. AI-driven analytical systems rely on large volumes of digital data and automated decision-making processes that raise important concerns related to transparency, accountability, and fairness. Scholars have emphasized the need for ethical frameworks capable of guiding the responsible development and deployment of artificial intelligence systems in healthcare and public health (18).

Concerns related to algorithmic bias further illustrate the importance of governance mechanisms capable of ensuring fairness and equity in digital health systems. Studies examining the use of algorithmic decision-support tools in healthcare have shown that poorly designed algorithms may reproduce existing social inequalities or generate unintended discriminatory effects if not carefully monitored (20). Similarly, research in health informatics has demonstrated that digital health interventions may inadvertently exacerbate existing disparities if technological systems are not designed with equity considerations in mind (21).

These ethical considerations are increasingly reflected in international policy initiatives aimed at establishing governance frameworks for trustworthy artificial intelligence. Organizations such as the World Health Organization and the European Commission have developed guidelines emphasizing the importance of transparency, accountability, human oversight, and ethical responsibility in the development of AI systems used within health contexts (17, 22). These principles are particularly relevant for digital public health systems that rely on large-scale analysis of digital communication data.

Within this evolving policy landscape, the conceptual framework developed in the present study provides a governance-oriented perspective on the integration of artificial intelligence within public health systems. By conceptualizing infodemic management as a socio-technical governance system, the framework highlights the need for coordinated interaction between digital data ecosystems, AI-driven analytical tools, human expertise, and institutional decision-making mechanisms.

The framework also contributes to the growing field of digital public health, which examines the ways in which digital technologies can support population health interventions and strengthen public health systems (23). Digital public health approaches emphasize the importance of integrating technological innovation with governance structures capable of ensuring that digital tools are used effectively and responsibly within health systems.

Importantly, the framework underscores the continued importance of human expertise in interpreting algorithmic outputs and translating digital signals into actionable knowledge. While artificial intelligence technologies can identify patterns within complex datasets, human interpretation remains essential for contextualizing analytical findings within epidemiological, social, and political contexts. Public health decision-making therefore requires collaborative interaction between technological systems and expert judgment.

This role becomes particularly important when AI systems encounter information that is context-dependent, ambiguous, or partially accurate. Misinformation frequently appears in the form of selective truths, misleading framing, or evolving narratives that may not be reliably classified through automated approaches alone. Consequently, human oversight functions as a critical safeguard against algorithmic misjudgments and contributes to maintaining the reliability of digital public health intelligence systems. In addition, expert review can generate high-quality annotated examples that may be reintegrated into machine learning pipelines, supporting iterative model improvement and enhancing the contextual sensitivity of future analyses.

Taken together, the findings of this study suggest that effective responses to infodemics require governance models capable of integrating digital surveillance systems, artificial intelligence analytics, interpretive expertise, and institutional decision-making processes. Future research should explore the operational implementation of such governance models within public health institutions and examine how digital intelligence systems can be integrated into existing surveillance and policy frameworks. Future empirical studies should evaluate the framework using measurable indicators including detection timeliness, algorithm accuracy, false-alert burden, intervention effectiveness, behavioural outcomes, public trust, equity indicators, and unintended consequences.

### Implications for digital public health governance

The findings of this study have several implications for the development of governance strategies aimed at strengthening public health responses within increasingly complex digital communication environments. As digital platforms continue to reshape the circulation of health information, public health systems must adapt their surveillance, communication, and decision-making mechanisms in order to respond effectively to emerging infodemic dynamics.

First, the framework proposed in this study highlights the importance of integrating digital data ecosystems into public health intelligence systems. Traditional surveillance infrastructures have historically focused on epidemiological indicators derived from clinical and laboratory data. However, digital communication platforms generate vast quantities of real-time information reflecting public perceptions, attitudes, and behavioral responses to health risks. Incorporating digital data streams into public health monitoring systems can therefore enhance situational awareness during health crises and allow institutions to detect emerging misinformation narratives at earlier stages (5, 15).

Second, the increasing availability of artificial intelligence technologies creates new opportunities for strengthening digital public health surveillance capabilities. Machine learning algorithms and natural language processing techniques enable large-scale analysis of unstructured communication data and can support the detection of misinformation patterns across complex digital networks (7). However, the effective use of these technologies requires governance structures capable of integrating algorithmic outputs with institutional decision-making processes. Without appropriate governance mechanisms, digital surveillance systems risk generating large volumes of analytical information without producing meaningful policy responses.

Third, the framework emphasizes the central role of human expertise in interpreting algorithmic insights generated through artificial intelligence systems. While computational methods are highly effective in identifying patterns within large datasets, the interpretation of these signals requires contextual knowledge derived from epidemiology, behavioral sciences, and risk communication research. Public health professionals therefore remain essential actors within digital surveillance systems, serving as intermediaries between technological analysis and institutional decision-making processes.

Fourth, the governance of digital public health systems must incorporate ethical and regulatory safeguards capable of ensuring transparency, accountability, and equity in the use of artificial intelligence technologies. Scholars have emphasized that algorithmic systems used within health contexts must operate within ethical frameworks that protect individual rights and minimize the risk of discriminatory outcomes (18, 19). Concerns regarding algorithmic bias and digital inequalities further highlight the need for governance mechanisms capable of ensuring fairness and inclusiveness in the deployment of AI-driven health technologies (20, 21).

Finally, the framework suggests that effective infodemic management requires a multilevel governance approach capable of coordinating actions across public health institutions, digital platforms, research organizations, and policy authorities. The transnational nature of digital communication environments means that misinformation can spread rapidly across national borders, requiring coordinated responses that extend beyond individual public health agencies. International organizations and regulatory bodies therefore play an important role in developing shared governance principles and collaborative strategies aimed at strengthening digital resilience within public health systems.

Taken together, these implications suggest that the future of public health governance will increasingly depend on the ability of institutions to integrate digital intelligence, artificial intelligence technologies, and coordinated policy responses within adaptive governance frameworks capable of responding to rapidly evolving information environments.

### Illustrative application scenario

To illustrate the operational logic of the framework, consider a hypothetical outbreak characterized by the rapid dissemination of misleading information regarding vaccine safety across social media platforms. Within the data ecosystem layer, digital surveillance systems identify unusual increases in specific keywords, hashtags, and online discussions associated with vaccine-related concerns. AI-driven analytics subsequently classify emerging narratives, monitor sentiment trends, and detect patterns suggestive of coordinated misinformation activity.

The resulting outputs are reviewed within the interpretation and translation layer by public health professionals, epidemiologists, and communication specialists. Through contextual assessment and evidence verification, experts distinguish legitimate public concerns from misleading or inaccurate claims and evaluate their potential public health implications.

Validated findings are then transferred to the decision and policy layer, where public health authorities develop targeted communication campaigns, coordinate messaging strategies with trusted stakeholders, and implement evidence-informed interventions aimed at mitigating misinformation and restoring public trust. Simultaneously, expert evaluations of AI-generated outputs are incorporated into model refinement processes, supporting continuous learning and performance improvement.

This hypothetical example illustrates how the framework may facilitate the transformation of digital information signals into coordinated institutional responses within public health systems.

Within this operational process, predefined decision thresholds should determine whether the detected signal warrants institutional intervention, taking into account the credibility of the information source, the level of algorithmic uncertainty, the potential public health impact, and the likelihood of false-positive or false-negative classifications. Responsibility for the final decision remains with the competent public health authority, ensuring that AI-generated outputs function as decision-support tools rather than autonomous decision-makers. Following implementation, intervention outcomes—including dissemination trends, public engagement, trust indicators, equity considerations, and behavioural responses—should be systematically monitored to evaluate effectiveness and to support continuous refinement of both governance procedures and AI analytical models.

### Limitations

A further limitation concerns the absence of formal framework validation. The present study was designed as a conceptual and theory-generating investigation aimed at developing an evidence-informed governance model. Consequently, the framework has not yet undergone expert consensus procedures, Delphi processes, stakeholder assessment, or empirical implementation testing. Future research should evaluate the validity, feasibility, and applicability of the proposed model through structured validation approaches involving public health professionals, policymakers, digital health experts, and implementation studies conducted in real-world settings.

Several limitations should be considered when interpreting the findings of this study. First, the framework proposed in this work is conceptual and has not yet been empirically validated through implementation studies within public health institutions. Conceptual frameworks provide analytical models capable of guiding research and policy development, but their practical effectiveness must be assessed through future empirical studies examining how such governance models operate in real-world public health systems.

Second, the evidence base informing the framework derives primarily from studies examining digital information dynamics during recent global health crises, particularly the COVID-19 pandemic. While this context provided valuable insights into the mechanisms through which misinformation spreads across digital communication ecosystems, the rapid evolution of digital technologies and communication platforms may influence the ways in which infodemics develop in future public health emergencies.

Third, the analytical focus of this study is centered on digital information ecosystems and artificial intelligence–driven analytical tools. Although these technologies provide powerful capabilities for monitoring complex information environments, effective responses to infodemics also depend on broader social, cultural, and political factors that extend beyond technological solutions. Public trust in health institutions, media literacy levels, and the broader political context can all influence how misinformation spreads and how populations respond to public health communication strategies.

Finally, the conceptual framework emphasizes governance structures capable of integrating digital analytics into public health decision-making processes. However, institutional capacities, technological infrastructures, and regulatory environments vary substantially across countries and health systems. As a result, the implementation of such governance models may require adaptation to different institutional and socio-political contexts.

Despite these limitations, the framework offers an analytical perspective that may support the understanding of how digital information environments, artificial intelligence analytics, expert interpretation, and institutional governance interact in shaping public health responses to infodemics. Future research should therefore focus on empirically testing and operationalizing the proposed model across different public health systems in order to assess its applicability and its potential contribution to strengthening infodemic preparedness and digital health governance.

Furthermore, because framework development involved interpretive conceptual synthesis, some degree of researcher judgement was unavoidable despite the consensus-based analytical process adopted by the research team.

## Conclusion

The increasing complexity of digital communication ecosystems has fundamentally transformed the landscape of public health information environments. In this context, the management of infodemics requires not only technological tools capable of monitoring digital information flows but also governance structures able to translate digital intelligence into coordinated public health action.

This study proposed a conceptual governance framework integrating four key components—digital data ecosystems, artificial intelligence analytics, interpretive public health expertise, and institutional governance mechanisms—within a unified socio-technical model. By synthesizing evidence from the existing literature, the framework highlights how digital signals generated within online communication environments may be progressively transformed into actionable knowledge capable of informing institutional responses to misinformation.

The proposed model contributes to the literature by shifting the focus from purely technological solutions toward governance-oriented approaches to digital public health. While artificial intelligence can enhance the capacity to detect and analyze information dynamics within digital ecosystems, effective responses to infodemics ultimately depend on institutional arrangements capable of integrating analytical insights with policy development, communication strategies, and coordinated public health interventions.

Although the framework remains conceptual and requires empirical validation, it provides a structured analytical lens for examining the interaction between digital technologies, expert interpretation, and institutional decision-making processes in the management of health misinformation.

Future research should focus on testing and operationalizing the framework in real-world public health systems, exploring how artificial intelligence–driven insights can be effectively integrated into governance structures, decision-making processes, and risk communication strategies during public health crises.

Strengthening governance capacity within digital information environments will therefore be essential for improving the resilience of public health systems in the face of future infodemics. By clarifying how digital intelligence, artificial intelligence analytics, expert interpretation, and institutional governance can interact within an integrated decision-making architecture, the framework proposed in this study offers a conceptual foundation for future empirical research and policy development in digital public health governance.

Furthermore, the framework relies on evidence synthesized from a single scoping review and therefore reflects the characteristics of that evidence base, including its temporal search window and the predominance of studies conducted during the COVID-19 pandemic. The framework should therefore be regarded as an evidence-informed conceptual governance model requiring subsequent empirical validation, stakeholder evaluation, and operational implementation studies before routine adoption within public health systems.

## Data Availability

The original contributions presented in the study are included in the article/Supplementary Material, further inquiries can be directed to the corresponding author/s.
